# The Protective Effects of Silymarin against Doxorubicin-Induced Cardiotoxicity and Hepatotoxicity in Rats

**DOI:** 10.3390/molecules16108601

**Published:** 2011-10-12

**Authors:** Aleksandar Rašković, Nebojša Stilinović, Jovanka Kolarović, Velibor Vasović, Saša Vukmirović, Momir Mikov

**Affiliations:** 1 Department of Pharmacology, Toxicology and Clinical Pharmacology, School of Medicine, University of Novi Sad, 21000 Novi Sad, Serbia; Email: araskovic@hotmail.com (A.R.); vasovicv@neobee.net (V.V.); sasavukmirovic99@gmail.com (S.V.); momir.mikov@otago.ac.nz (M.M.); 2 Department of Hematology and Oncology, Institute for Child and Youth Health Care of Vojvodina, Novi Sad, 21000 Novi Sad, Serbia; Email: jovanka.kolarovic@gmail.com (J.K.)

**Keywords:** silymarin, silibinin, doxorubicin, cardiotoxicity, hepatotoxicity

## Abstract

Silymarin is a complex of five major compounds, and silibinin is the most biologically active component of the complex. The aim of this study was to investigate, evaluate and confirm the potential cardioprotective and hepatoprotective effects of administration of silymarin, rich in silibinin, at a dose of 60 mg/kg orally for a time-span of 12 days on doxorubicin induced toxicity in male Wistar rats. The *in vivo* model was used to explore whether silymarin could prevent damage of liver and heart tissue induced by doxorubicin administered every other day at dose of 1.66 mg/kg intraperitoneally for twelve days. In the study the change of body weight, ECG changes, biochemical parameters of oxidative stress, serum activity of alanine and aspartate transaminase, lactate dehydrogenase, creatine kinase and histological preparations of heart and liver samples of treated animals were examined. According to physiological, pharmacological, microscopic and biochemical results, we confirmed that at the examined dose, silymarin exhibits a protective influence on the heart and liver tissue against toxicity induced by doxorubicin.

## 1. Introduction

We are witnessing a growing popularity of complementary and alternative medicine (CAM), primarily herbal medicine. It is associated with an ongoing debate of integrating it into mainstream healthcare, but the acceptance of CAM among physicians has increased in the last years [1].

Silymarin is one of the most successful examples of development of a modern drug from a complementary and alternative medicine. Silymarin is isolated from *Silybum marianum* L. Gaertn. herb, also known as, Mary thistle and milk thistle in English or Mariendistel in German. Milk thistle belongs to the Asteraceae (Compositae) family, one of the largest families of plants. As a wild plant, it used to grow in Europe and Asia only, but nowadays it is regularly seen in North and South America, partially cultivated, in order to be exploited and used for therapeutical purposes, and partially as a wild plant, renewing the cycle of its existence due to its random seed transfer [2,3].

Milk thistle has been used in Europe for centuries for hepatic and biliary disorders. It is used as an antidote for *Amanita* mushroom poisoning and to protect the liver and kidneys from toxic medications. The German Commission E recommends it for the treatment of dyspeptic complaints, toxin-induced liver damage, and hepatic cirrhosis and as a supportive therapy for chronic inflammatory liver conditions [4,5,6].

Silymarin is a complex of five major compounds, including four flavonolignans: Silibinin (silybin A, silybin B, isosilybin A and isosilybin B), silychristin, isosilychristin, silydianin, and one flavonoid, taxifolin [7,8]. Silymarin is used as supportive treatment for liver diseases of different etiology where it is hepatoprotective through its antioxidant activity, stimulates protein synthesis, influences lipid metabolism, and stabilizes membrane phospholipids. Silymarin reacts with the reactive oxygen species (ROS) and converts them into less reactive and toxic compounds. It potentiates the effects of the physiological antioxidants (glutathione, superoxide dismutase) and prevents the reduction of their concentrations, as well as its structural and functional consequences [9,10,11]. *In vitro* and animal data suggest that silymarin may increase the uptake and actions of chemotherapeutic agents. Preclinical investigations have found that silymarin increased daunomycin accumulation, potentiated doxorubicin activity, and inhibited efflux of these drugs from cancer cells [12,13].

Silibinin is the most biologically active component of the complex, with a distinct hepatoprotective activity [7,8,14]. Silibinin has been reported to exert antioxidative and free radical scavenging abilities. Its iron chelator activity was reported [10,15]. Recent studies showed that silibinin modulates the imbalance between cell survival and apoptosis through the regulation of cell cycle regulators, which can help the therapy of various cancers [16]. Silibinin also potentiated the antitumor action of cisplatin *in vivo* and *in vitro* [11,17].

Studies have indicated enhancement of anticancer therapy by silymarin, but there is a little data showing that silymarin can prevent the toxicity of the most commonly used anticancer agents. One of the most commonly used antineoplastic agents is doxorubicin. Doxorubicin, an anthracycline antibiotic, is a broad spectrum antineoplastic agent, used for the treatment of uterine, ovarian, breast and lung cancers, Hodgkin’s disease and soft tissue sarcomas as well as for several other cancer types. It is believed that oxidative stress and the formation of free radicals, which also involves a reaction of doxorubicin with iron, play a crucial role in the mechanism of doxorubicin toxicity [18,19,20,21]. Doxorubicin is significantly toxic to most tissues and organs, but its cardio and hepatotoxicity are limiting factors in the cancer therapy with this agent. Many researchers have tried to find ways to reduce the adverse effects associated with doxorubicin therapy. There are promising preclinical results in protecting against doxorubicin-induced toxicity through application of different natural products, such as celery and parsley juices, catechin, fullerenol C_60_(OH)_24_ and others [17,18,19,20,21,22]. Nevertheless, it must be pointed out that most studies have investigated the influence of a high single dose of doxorubicin on the heart and liver tissue, while only a few of them are dealing with evaluation over several weeks.

The hypothesis proposed was that if doxorubicin cardiotoxicity and hepatotoxicity are related to free radical formation and oxidative stress, an antioxidant such as silymarin may protect against doxorubicin-induced toxicity in the heart and liver. Therefore, the aim of this study was to investigate, evaluate and confirm the potential cardioprotective and hepatoprotective effects of silymarin, rich in silibinin, in rats after a multi-dose administration of doxorubicin.

## 2. Results and Discussion

### 2.1. Change in the Body Weight

It can be seen from Table 1 that the greatest increase in body weight was among the animals that were treated with olive oil only (ConO), while the greatest decrease in body weight was observed in the experimental group treated with doxorubicin (Dox), as expected. In the experimental group treated with a combination of doxorubicin and silymarin has was also a decrease in body weight, but the loss was statistically significantly smaller in comparison with the experimental group treated with doxorubicin only.

**Table 1:** Change in the body weight (Δ) of the animals of control saline (ConS), control olive oil (ConO) and experimental groups treated with silymarin (Sil), doxorubicin (Dox) and the combination of doxorubicin and silymarin (DoxSil).

Animal groups	ConS	ConO	Sil	Dox	DoxSil
**Δ Body Weight (g)**	6.67 ± 1.63	16.67 ± 4.23 ^a^	10.83 ± 3.76 ^b^	−27.00 ± 7.64 ^a^	−15.83 ± 3.54 ^c^

^a^ p < 0.001 compared to ConS; ^b^ p < 0.05 compared to ConS; ^c^ p < 0.01 compared to Dox; n = 6; mean ± SD.

This experiment confirmed that the use of doxorubicin leads to a significant drop in body weight of experimental animals [18,19]. By comparing results of experimental groups it can be assumed that treatment with the silymarin prevented greater body weight loss induced with doxorubicin. As the greatest increase in body weight was observed among animals treated with olive oil and since silymarin was dissolved in olive oil, its effect on the reduction of loss of body weight DoxSil in the experimental group cannot be confirmed with certainty.

### 2.2. Evaluation of ECG Changes

Doses of lidocaine required to cause bradycardia in ECG record are statistically significantly higher in the Dox group compared to the control groups (ConS and ConO) and experimental groups Sil and DoxSil (Table 2).

**Table 2:** Dose of lidocaine, administered through continuous intravenous infusion, required for causing bradycardia in control saline (ConS) and control oil (ConO), and experimental groups treated with silymarin (Sil), doxorubicin (Dox) and the combination of doxorubicin and silymarin (DoxSil).

Animal groups	ConS	ConO	Sil	Dox	DoxSil
**Lidocaine dose (mg/kg)**	15.36 ± 0.68	13.87 ± 2.53	16.33 ± 1.20	26.33 ± 2.65 ^a^	19.01 ± 0.61 ^b^

^a^ p < 0.01 compared to ConS, ConO, Sil and DoxSil; ^b^ p < 0.05 compared to ConS, ConO and Sil; n = 6; mean ± SD.

Harmful effects of doxorubicin on myocardial function are reflected in the morphological and functional changes of cardiomyocytes, proarrhythmogenic effect and in causing irreversible heart failure. The proarrhythmogenic effect of the anthracycline antibiotics is manifested as acute or subacute transient ventricular or supraventricular arrhythmia. The most common electrophysiological disorders are in form of non-specific changes in ST-segment and T-waves, reducing in voltage of QRS complex, QT-wave prolongation, sinus or ventricular tachycardia, and supraventricular or ventricular extrasystole [23,24]. There are studies showing the substances that can help preventing proarrhythmogenic effect of doxorubicin [19,23,25].

In our investigational model, treatment with doxorubicin led to an increase in myocardial excitability, which is reason why it took significantly more lidocaine to cause bradycardia in the Dox group compared to the control, Sil and DoxSil groups. In the group of experimental animals that were treated with a combination of doxorubicin and silymarin significantly lower doses of lidocaine were required to cause bradycardia, and therefore silymarin prevented increase in myocardial excitability caused by doxorubicin.

### 2.3. Biochemical Parameters of Rat Liver Homogenate

The process of lipid peroxidation, was statistically significantly higher in group treated with doxorubicin, compared to the control groups ConS and ConO and experimental groups Sil and DoxSil. Xanthine oxidase and catalase enzyme activity in the experimental group treated with doxorubicin is statistically significantly higher compared to the control groups and experimental group treated with silymarin and combination of doxorubicin and silymarin.

There is no statistically significant difference in the concentration of reduced glutathione between the control and the experimental groups. Applied separately, doxorubicin led to a statistically significant increase in glutathione peroxidase activity, in comparison with the enzyme activity of the animals of control groups and experimental groups treated with silymarin and combination of doxorubicin and silymarin (Table 3).

**Table 3:** Parameters of oxidative stress in livers of rats treated with saline, olive oil, silymarin, doxorubicin and combination of silymarin and doxorubicin.

	ConS	ConO	Sil	Dox	DoxSil
**LPx**	8.8 ± 0.6	10.05 ± 1.8	11.4 ± 1.9	22.5 ± 2.8 ^a^	15.6 ± 1.1
**XOD**	12.55 ± 0.80	12.14 ± 0.34	11.13 ± 0.92	14.22 ± 0.75 ^a^	12.58 ± 0.79
**CAT**	3.45 ± 0.60	3.54 ± 0.81	4.12 ± 0.42	5.98 ± 0.40 ^a^	4.52 ± 0.31
**GSHPx**	3.13 ± 0.23	4.38 ± 0.67	2.72 ± 0.3^b^	5.30 ± 0.45 ^a^	3.501 ± 0.25
**GSH**	31.50 ± 3.57	30.87 ± 1.63	29.72 ± 3.12	28.60 ± 2.23	28.45 ± 1.85

^a^ p < 0.05 compared to ConS, ConO, Sil and DoxSil; ^b^ p < 0.05 compared to ConO and Dox; n = 6; mean ± SD. GSH is expressed in nmol/mg of protein, activity of XOD, GSHPx and CAT are expressed in nmol/mg of protein min^−1^. The intensity of lipid peroxidation (LPx) is expressed in nmol malondialdehyde/mg of protein.

Doxorubicin is a very potent antitumor antibiotic. Its use is severely limited for its cardiotoxicity and hepatotoxicity, which have been documented in a variety of animal models [9,10,20,21]. The semiquinone form of doxorubicin is a toxic short-lived metabolite which interacts with molecular oxygen and initiates a cascade of reactions, producing reactive oxygen species (ROS). ROS generation and lipid peroxidation have been suggested to be responsible for doxorubicin-induced cardio and hepatotoxicity [9,10,18,19].

In experiments on human subjects, has been observed that silymarin significantly increases antioxidant capacity of tissue, and thus, may explain the cardioprotective effect of silymarin [26]. There are also *in vitro* and studies in animals showing antioxidant properties of silymarin [8,9,10,14].

Silymarin reacts with cell membranes and increases their resistance to harmful influences, most likely through changes in their physicochemical properties, but also interacts with ROS and converts them into less reactive and toxic compounds. In various studies it was confirmed that silymarin prevents lipid peroxidation and increases the concentration of glutathione [18,19,20,21].

Our experiment confirmed these effects of silymarin. Treatment with silymarin led to a significant decrease in the process of lipid peroxidation in comparison to the experimental group that received only doxorubicin. This study did not reveal any significant differences in the concentrations of reduced glutathione between the experimental groups. However, there has been a slight decrease in glutathione concentration, in the group of animals treated with doxorubicin in comparison with the ConS, ConO and Sil groups. A significant difference was observed by comparing the activity of glutathione peroxidase enzyme, where the treatment with silymarin has led to a reduction in glutathione peroxidase activity in the Sil and DoxSil groups of animals. Similar differences were noticed by observing the results of activity of the catalase enzyme, which was significantly increased due to the effects of doxorubicin. Because doxorubicin makes ROS in organism, catalase enzyme becomes more reactive and decomposes them into nontoxic molecules, water and oxygen [27].

Xanthine oxidase enzyme can be normally found in the liver and it is important in the metabolism of purines. In the chemical reactions that are catalyzed by xanthine oxidase, side product is hydrogen peroxide, which belongs to ROS. A study by Dawson and Walters pointed out that inhibition of xanthine oxidase can be beneficial in the prevention of cardiovascular diseases [28]. In our study, XOD activity was the lowest in the group of animals treated with silymarin and silymarin also prevented doxorubicin-induced increase of XOD.

### 2.4. Serum Enzymes Activity

There are no statistically significant differences in the alanine transaminase activity between the groups.

Aspartate aminotransferase activity was statistically significantly increased in rats treated with doxorubicin compared to the groups of animals treated with saline only, silymarin only and combination of doxorubicin and silymarin. Concentration of lactate dehydrogenase and creatine kinase is significantly higher in the serum of animals treated with doxorubicin only than in the other groups (Table 4).

**Table 4:** Serum enzymatic findings (U/L), in rats treated with saline, olive oil, silymarin, doxorubicin and combination of silymarin and doxorubicin.

	ALT	AST	LDH	CK
**ConS**	40.03 ± 3.4	270 ± 85.7	998 ± 129	316 ± 104
**ConO**	43.3 ± 5.8	299 ± 131.74	1012 ± 215	323 ± 76
**Sil**	49.8 ± 3.5	198.8 ± 32.69	946 ± 117	279 ± 89
**Dox**	60 ± 25.5	349.6 ± 71.5 ^a^	2325 ± 343 ^c^	616 ± 39 ^b^
**DoxSil**	58.5 ± 12.4	279.5 ± 47.5	1843 ± 487 ^d^	402 ± 81

^a^ p < 0.05 compared to ConS, Sil and DoxSil; ^b^ p < 0.01 compared to ConS, ConO, Sil, DoxSil; ^c^ p < 0.01 compared to ConS, ConO and Sil; ^d^ p < 0.05 compared to Sil; n = 6; mean ± SD.

It was previously known that heart is very sensitive to ROS induced damage because of its highly oxidative metabolism and fewer antioxidant defenses compared to other organs, so an increase of LDH and CK concentration in the serum of animals treated with doxorubicin is expected. In this study, it was found that multi-dose administration of doxorubicin induced marked cardiotoxicity. Our results were in agreement with reports of other authors [9,19]. The results of the present study also revealed that silymarin pretreatment attenuated doxorubicin-induced cardiotoxicity. This was manifested by the significant decreased serum CK and LDH activities.

The research results of other authors have pointed to the fact that doxorubicin did not show adverse effects at the myocardial level only, but also the possibility of significant damage of liver function, and consequent increase in serum transaminase activity [18,19,29]. Our study confirmed that doxorubicin causes changes in aspartate aminotransferase activity, but that the changes can be prevented if doxorubicin is applied together with silymarin [29].

### 2.5. Evaluation of Histological Changes

**Figure 1:**
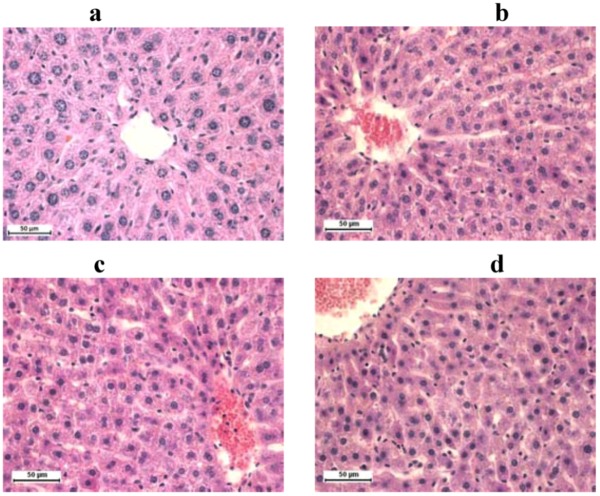
Rat liver stained with H&E technique, 400× magnification: (**a**) control groups; (**b**) group treated with silymarin; (**c**) doxorubicin; (**d**) and combination of doxorubicin and silymarin.

Images of histological preparations show that hepatocytes seem usual, with visible nuclei. In the portal area biliary ducts can be seen, also looking normally, without any signs of cholestasis. There are no pathological changes in histological preparations of the liver tissue in ConS and ConO group of animals (Figure 1a). Mild to moderate hyperemia was present in the liver tissue of all experimental animals (Figure 1b–d).

**Figure 2:**
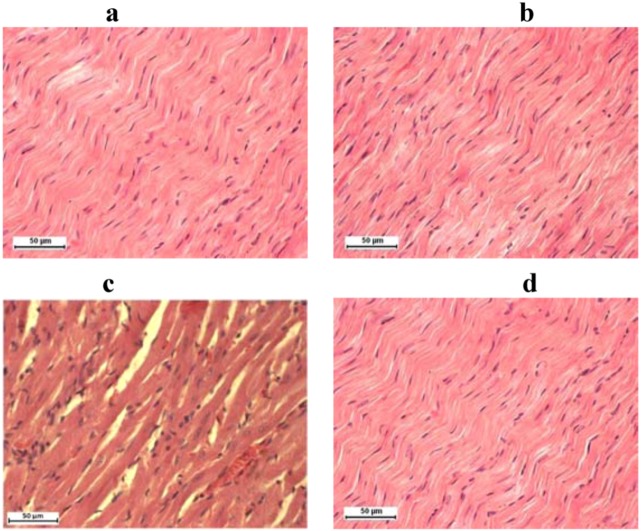
Rat heart stained with H&E technique, 400× magnification: (**a**) control groups, (**b**) group treated with silymarin, (**c**) doxorubicin, (**d**) and combination of doxorubicin and silymarin.

In histological preparations of the heart of animals treated with saline only, and only with olive oil, silymarin only and combination of doxorubicin and silymarin there is a morphological structure that corresponds to physiological appearance of the organ. Cardiomyocytes are regular, cylindrical, with no visible signs of degeneration or necrosis. Between cardiomyocytes is endomysium in normal quantities, with no signs of fibrosis (Figure 2a, 2b and 2d). In the group of animals that was treated with doxorubicin (Dox) the only visible pathological sign is moderate hyperemia (Figure 2c).

Histological examination by light microscopy can prove anthracycline toxicity, but it can only reveal gross cellular changes. By using electronic microscopy more common ultrastructural changes such as sarcotubular edema and disorders of the fine mitochondrial structure can be found [30].

In our study the structural and functional changes, demonstrated microscopically in the heart and liver tissue of doxorubicin intoxicated rats, were mild. Although various authors have demonstrated lesions, histologically classified as focal cytoplasmic vacuole formation, circumscribed areas of myofibrillar loss of cardiomyocytes and single cell necrosis and parenchymal degeneration of hepatocytes, we have only found hyperemia as pathological sign. Their reports have shown that supplementation with antioxidants in doxorubicin-induced myocardial and hepatic injury has a favourable effect [19,22,31].

We have found functional changes in heart and liver tissue by ECG and biochemistry assays, although histological examination by light microscopy did not show significant cell degeneration or necrosis caused by doxorubicin. Significant hyperemia only in the group of animals treated with doxorubicin, but not in the group treated with combination (DoxSil), can point to protective role of silymarin and confirm other findings from our study.

## 3. Experimental

### 3.1. Animals

Experiments were carried out on adult sexually mature male Wistar rats, weighing 250–300 g and ages up to three months, which were bred at the Department of Pharmacology, Toxicology and Clinical Pharmacology at the Faculty of Medicine in Novi Sad, Serbia. Laboratory animals were under human care in accordance with the criteria given in the “Guide for the Care and Use of Laboratory Animals” edited by Commission of Life Sciences, National Research Council (USA). The study was approved by the Ethics Committee of the University of Novi Sad. Laboratory animals were quarantined and housed in Ehret UniProtect Air Flow Cabinet with High-Efficiency Particulate Air (HEPA) filter system (EHRET GmbH und Co, Germany), three per standard plexiglass cage at a controlled 21 ± 1 °C temperature and 55% ± 1.5% humidity with standard circadian rhythm (day/night). They had free access to a standard laboratory diet (Veterinary Institute, Zemun, Serbia) and water. The animals were randomly divided into test and control groups, each group consisting of eight animals.

### 3.2. Chemicals

We used a commercial product of silymarin (rich in silibinin) called Silymarin-CT capsules, made by CT Arzneimittel GmbH pharmaceutical company (Berlin, Germany). They contain silymarin extracted from milk thistle seeds by ethanolic extraction. Each capsule contained 117 mg of silymarin, calculated as silibinin and standardized according to DAB_2001_ HPLC method. Content of the capsules was fragmented in mortar and dissolved in olive oil (Oleum Olive, Ph. Yug. V). Due to its poor water solubility we have chosen a lipid solvent to enhance absorption of silymarin in the gastrointestinal tract [14,23]. Doxorubicin (Doxorubicin “EBEWE”) was obtained from “EBEWE Pharma GmbH” (Unterach, Austria) as concentrate for solution. The solution for the intraperitoneal (i.p.) application was dissolved in a sterilized and apyrogenic 0.9% NaCl solution (2 mg/mL) inside a laminar flow cabin. Lidocaine was purchased from “Galenika a.d” (Belgrade-Zemun, Serbia). For the narcotization of animals we used 25% solution of urethane (Sigma Chemicals Co, St Louis, MO, USA).

### 3.3. Experimental Treatment

In order to perform the experiment we used 30 animals divided into five groups. All animals were weighed before the experiment and after the 12-day treatment period. The first control group was treated with saline at a dosage of 1 mL/kg TM orally (ConS), while the second control group received olive oil at a dosage of 1 mL/kg orally (ConO). By reviewing the available literature we determined the dose of doxorubicin and silymarin for experiment [18,19,20,21,22,32,33,34]. Experimental groups were treated with silymarin (Sil) every day at a dosage of 60 mg/kg orally, doxorubicin (Dox) every second day at a dosage of 1.66 mg/kg i.p. and with the combination of doxorubicin and silymarin (DoxSil) in stated doses. Peroral application of previously mentioned substances was by using oral gavage feeding needle. Treatment of the animals with the above mentioned substances lasted 12 days.

### 3.4. Evaluation of ECG Alterations

On the twelfth day, two hours after the application of the investigated substances, the animals were anesthetized with urethane at a dosage of 0.75 g/kg intraperitoneally and prepared for the execution of the electrophysiological part of the experiment. After the preparation of the jugular vein, subcutaneous needle electrodes of the commercial computer-based ECG device (Innomed, Budapest, Hungary) were connected to the animals, and electrocardiogram was recorded, initially without the influence of the tested cardioactive substance. Afterwards, cannula was introduced in jugular vein, through which the lidocaine was administered by infusion pump at a dosage of 10 mg per h. The effect was continously observed in the ECG record. Based on the duration of infusion, we calculated a dose of lidocaine required to induce bradycardia in the experimental animals. Dose of lidocaine is expressed in relation to body mass (mg/kg). Recording of bradycardia in the electrocardiogram was sign for stopping the infusion pump.

### 3.5. Biochemical Assays

Immediately after stopping the infusion pump the animal’s chest was opened in order to access the left heart chamber. Then animals were sacrificed by cardiopunction and the samples of blood and liver were taken. The obtained serum was used for the analysis of enzymatic activity of alanine transaminase (ALT), aspartate transaminase (AST), lactate dehydrogenase (LDH) and creatine kinase (CK). The enzyme activity was measured by kinetic method “Randex”, with biocommercial kits, on Agilent 8453 UV/Vis spectrophotometer (Agilent Technologies, Palo Alto, CA, USA)

Liver homogenate was prepared from 1 g of liver tissue which was homogenized in a Potter homogenizer with TRIS-HCl sucrose buffered solution in a ratio 1:3 at 4 °C. The obtained homogenates were filtered and the biochemical parameters were determined: Concentration of the reduced glutathione (GSH), as well as the intensity of the lipid peroxidation (LPx) and the activity of catalase enzyme (CAT), xanthine oxidase (XOD) and glutathione peroxidase (GSHPx). All analyses were done in according with previously used protocols [21,35]. Extent of lipid peroxidation, LPx, was determined after Buege and Aust [36], peroxidase (Px) activity was measured after Simon *et al.* [37] and the effect of catalase (CAT) after Beers and Sizer [38]. Glutathione peroxidase (GSH-Px) activity was evaluated as described in Chin *et al.* [39], xanthine oxidase (XOD) after Bergmayer [40], and reduced glutathione content (GSH) after Kapetanović and Mieyal [41]. The total protein content was determined after Gornall *et al.* [42].

### 3.6. Histological Examination

After sacrificing the animals, we have also taken samples of liver and heart tissue for histological analysis. Samples were placed in 10% buffered formalin solution for tissue fixation. After paraffin molding, all tissue samples were serially sectioned at 3 µm, stained with standard hematoxylin-eosin (H&E) technique and evaluated blindly without prior knowledge of the treatment status of each animal. A Leica light microscope combined with a Leica DC 100 photo camera (Leica Microsystems GmbH, Germany) was used for cross section analysis (magnification 400×).

### 3.7. Statistical Method

The level of significance between the groups was assessed with the Student’s t-test for small independent samples using MedCalc 9.2.0.1 software [43]. All data are expressed as mean ± standard deviation (SD). A value of p < 0.05 was considered to be statistically significant.

## 4. Conclusions

Based on the results of our present study, it can be concluded that doxorubicin applied in a dose of 1.66 mg/kg every second day for 12 days, causes a significant reduction in body weight of treated animals and that silymarin prevents this body weight decrease in rats treated with doxorubicin. Treatment with silymarin prevents increase in AST and CK serum activity and myocardial excitability of rats caused by doxorubicin. It also significantly reduces doxorubicin-prooxidative activity and decreases histological changes in liver and heart tissue of animals treated with doxorubicin. Taken together, the data suggest that silymarin ameliorated doxorubicin-induced cardiotoxicity and protected against doxorubicin-induced hepatotoxicity in male Wistar rats.
